# Azithromycin inhibits IL-1 secretion and non-canonical inflammasome activation

**DOI:** 10.1038/srep12016

**Published:** 2015-07-08

**Authors:** Guido A. Gualdoni, Tilman Lingscheid, Klaus G. Schmetterer, Annika Hennig, Peter Steinberger, Gerhard J. Zlabinger

**Affiliations:** 1 Institute of Immunology, Center for Pathophysiology, Infectiology and Immunology, Medical University of Vienna, Vienna, Austria; 2 Department of Infectious Diseases and Pulmonary Medicine, Charité - Universitätsmedizin Berlin, Germany; 3 Department of Laboratory Medicine, Medical University of Vienna, Vienna, Austria

## Abstract

Deregulation of inflammasome activation was recently identified to be involved in the pathogenesis of various inflammatory diseases. Although macrolide antibiotics display well described immunomodulatory properties, presumably involved in their clinical effects, their impact on inflammasome activation has not been investigated. We compared the influence of macrolides on cytokine induction in human monocytes. The role of intracellular azithromycin-accumulation was examined by interference with Ca^++^-dependent uptake. We have also analysed the signalling cascades involved in inflammasome activation, and substantiated the findings in a murine sepsis model. Azithromycin, but not clarithromycin or roxithromycin, specifically inhibited IL-1α and IL-1β secretion upon LPS stimulation. Interference with Ca^++^-dependent uptake abolished the cytokine-modulatory effect, suggesting a role of intracellular azithromycin accumulation in the modulatory role of this macrolide. Azithromycin’s inhibiting effects were observed upon LPS, but not upon flagellin, stimulation. Consistent with this observation, we found impaired induction of the LPS-sensing caspase-4 whereas NF-κB signalling was unaffected. Furthermore, azithromycin specifically affected IL-1β levels in a murine endotoxin sepsis model. We provide the first evidence of a differential impact of macrolides on the inflammasome/IL-1β axis, which may be of relevance in inflammasome-driven diseases such as chronic obstructive pulmonary disease or asthma.

Macrolides are a group of antibiotics known to have a broad range of immune-modulating properties apart from their antimicrobial effects^1^,^2^. Different macrolides have proven effective in preventing exacerbations of chronic obstructive pulmonary disease (COPD)^3^,^4^,^5^,^6^ and non-cystic fibrosis bronchiectasis^7^, improving lung function in cystic fibrosis patients^8^ and in ameliorating the disease course in subgroups of asthma patients^9^. Intriguingly, these effects were reached in dose regimens considerably lower than used in antimicrobial chemotherapy. Therefore, immune-modulating properties are considered responsible for at least parts of their clinical effects.

Inflammasomes are large cytosolic multiprotein complexes mediating innate immune activation through the control of caspase activity in response to various stimuli^10^. This activation results in a variety of cellular adaptations including the induction of a programmed cell death termed “pyroptosis” which enhances microbial killing^11^, and most importantly, the maturation and cleavage of the pro-inflammatory cytokines IL-1α, IL-1β and IL-18. The first step in the production of these cytokines is induced by NF-κB activation which leads to the transcription of premature forms of the cytokines^12^. Next, inflammatory caspases are activated by specific stimuli and cleave the cytokines to generate their active forms. Recent findings have established a dual role of LPS in the induction of both steps of IL-1β maturation^13^. The human homologue of the murine caspase-11, the inflammatory caspase-4, was shown to be an intracellular sensor of LPS and is, therefore, regarded responsible for this second functionality of LPS^14^.

The activation of inflammasome signalling by pathogens is an innate defence mechanism leading to microbial clearance^15^; however, excessive inflammasome activation is equally detrimental for the host^16^. Deregulation of inflammasome signalling was shown to be involved in a variety of human conditions as autoimmune diseases^17^, graft-versus-host disease^18^, as well as type 2 diabetes^19^. Recently, this deregulation was identified to be involved in the pathogenesis of respiratory diseases as well^20^. Downstream molecules of the inflammasome-cascade such as IL-1α, IL-1β and IL-18 were found to be increased in patients with COPD^21^,^22^,^23^ and several experimental studies have substantiated the causative role of this pathway in cigarette-smoke induced inflammation^21^,^22^,^24^. Furthermore, compelling evidence exists for the involvement of the inflammasome in asthma pathogenesis^25^.

Respiratory illnesses constitute a major clinical application of macrolide antibiotics. However, although immune-modulating properties of macrolide antibiotics are well investigated, studies on the impact on inflammasome signalling are lacking. Furthermore, comparative studies on the effects of different macrolides on human immune cell function are rare, and in studies searching for favourable immune-modulating properties, the choice of which macrolide to use is based only on pharmacokinetic considerations. Therefore, we set out to analyse the effect of macrolides on inflammatory caspase activation and IL-1 secretion in human immune cells and compared the effects of the most prominent macrolide compounds on this pathway.

## Results

### Azithromycin specifically inhibits IL-1β secretion of human monocytes

**Figure 1:**
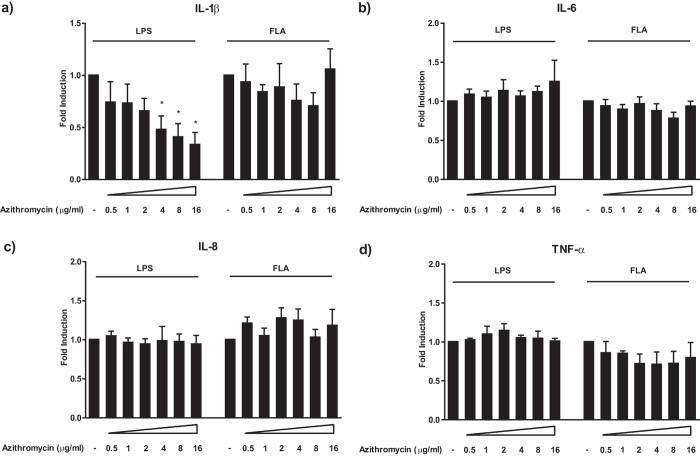
Azithromycin specifically inhibits IL-1β secretion upon LPS stimulation in human monocytes. Panel a)-d) show the impact of azithromycin on cytokine release of LPS or flagellin stimulated human monocytes. Cells were treated with the indicated concentrations of azithromycin and stimulated with either 100 ng/ml LPS or 100 ng/ml Flagellin for 20 h. After co-incubation, cell culture supernatants were analysed for the presence of the indicated cytokines by Luminex^®^. Values are expressed as mean ± SEM from 6 independent experiments. *Significant compared to LPS/flagellin alone, p < 0.05 calculated by paired t-test.

Since clinical evidence suggests beneficial effects of azithromycin on inflammasome-driven diseases^4^,^9^, we initially focused on this compound when analysing the impact of macrolides on pro-inflammatory cytokine release. In line with previous reports^26^,^27^, LPS stimulation alone was sufficient to induce a potent IL-1β release from human monocytes (Suppl. Table S1). Intriguingly, when stimulating human monocytes with LPS, azithromycin treatment significantly inhibited IL-1β secretion in a dose-dependent manner (Fig. 1a, Suppl. Table S1), whereas the induction of other monocyte-derived pro-inflammatory cytokines such as tumor necrosis factor (TNF)-α, IL-6, and IL-8 was unaffected (Fig. 1b–d, Suppl. Table S1). In contrast, when stimulated with flagellin, a TLR-5 ligand and an activator of the NLRC4 inflammasome, azithromycin treatment had no substantial effect on IL-1β secretion (Fig. 1a, Suppl. Table S1), and similar to LPS stimulation, the release of the other cytokines (TNF-α, IL-6, and IL-8) was not affected (Fig. 1b–d, Suppl. Table S1). Azithromycin in the doses used in these experiments did not exhibit significant effects on cell viability (Suppl. Fig. S1). These findings outline a specific and dose-dependent impact of azithromycin on the inflammatory caspase axis, leading to the inhibition of IL-1β maturation in human monocytes.

### Neither clarithromycin nor roxithromycin modulate cytokine production in human monocytes

**Figure 2:**
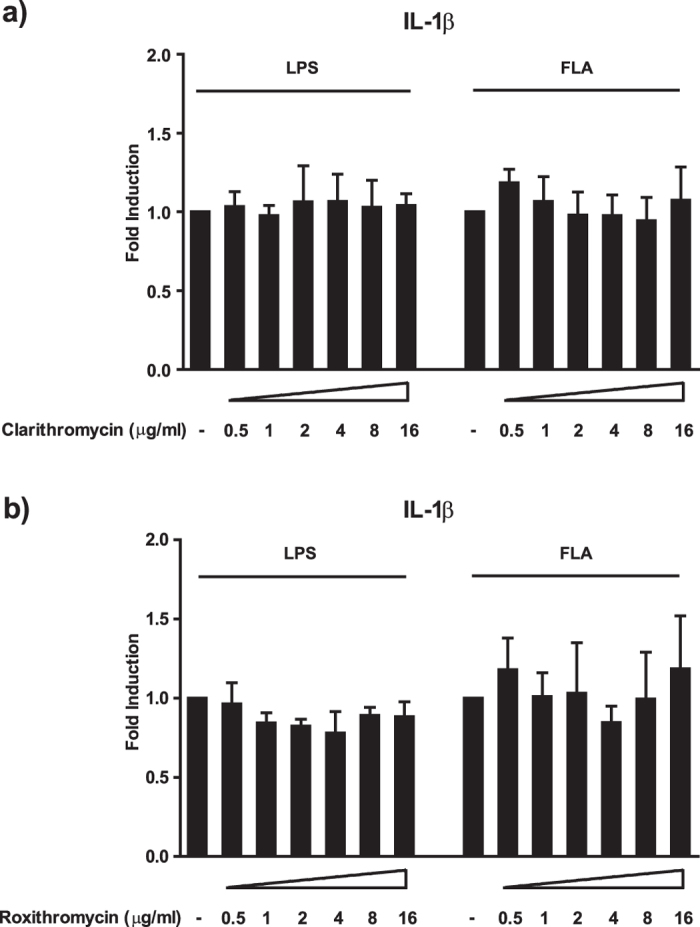
Clarithromycin and roxithromycin have no substantial impact on IL-1β secretion. Panel a) and b) show the impact of clarithromycin and roxithromycin, respectively, on LPS or flagellin stimulated IL-1β release by human monocytes. Cells were treated with the indicated concentrations of the drugs and stimulated with either 100 ng/ml LPS or 100 ng/ml flagellin for 20 h. After co-incubation, cell culture supernatants were analysed for the presence of the indicated cytokines by Luminex^®^. Values are expressed as mean ± SEM from 4 independent experiments.

Next, we wanted to find out whether other macrolides have an effect on IL-1β secretion in monocytes as well. For this purpose, we analysed the impact of clarithromycin and roxithromycin on monocytes stimulated with LPS or flagellin. In contrast to azithromycin, neither clarithromycin nor roxithromycin affected IL-1β secretion (Fig. 2a,b, Suppl. Table S1). Induction of the other pro-inflammatory cytokines was also not altered by treatment with these compounds (Suppl. Fig. S2, Suppl. Table S1). These experiments established a unique IL-1β modulating effect of azithromycin among the macrolides studied.

### Interference with azithromycin uptake abrogates its cytokine-modulating effect

**Figure 3:**
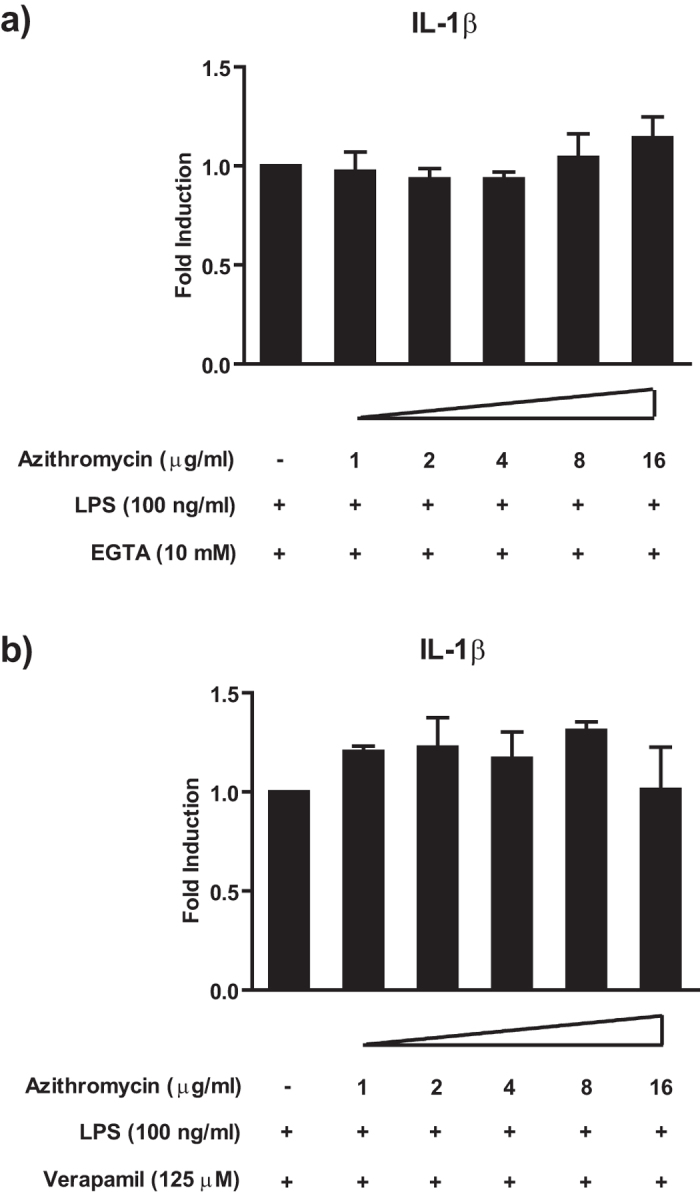
Interference with Ca^++^-dependent intracellular azithromycin accumulation abrogates the cytokine-modulating effect. Panel a) shows the impact of azithromycin on LPS stimulated human monocytes in the presence of 10 mM EGTA. Panel b) shows the impact of azithromycin IL-1β production of verapamil pretreated (30 min) and LPS stimulated monocytes in the presence verapamil. Cells were treated with the indicated concentrations of azithromycin and stimulated with 100 ng/ml LPS for 20 h. After co-incubation, cell culture supernatants were analysed for the presence of the indicated cytokines by Luminex^®^. Values are expressed as mean ± SEM from 3 independent experiments.

Azithromycin is known to accumulate in leucocytes to a considerably higher degree than any other macrolide antibiotic^28^,^29^. Drug transport through the plasma membrane (and thus intracellular accumulation) is Ca^++^-dependent and is assumedly operated via Ca^++^-channels^30^,^31^. We hypothesized that differences in intracellular accumulation were responsible for the diverging results observed in our study. To test this hypothesis, we disrupted the transmembrane transport of azithromycin by depleting extracellular calcium as well as utilising the L-type Ca^++^-channel blocker verapamil, two approaches shown to inhibit macrolide uptake in leucocytes^30^,^31^. As expected, both strategies interfering with azithromycin uptake resulted in the abrogation of its IL-1β modulating effect (Fig. 3a,b, Suppl. Table S1). These findings suggest a role of intracellular azithromycin accumulation in the modulatory effects of this macrolide which might be of relevance for the differential effects among macrolides.

### Azithromycin impairs intracellular LPS-sensing

**Figure 4:**
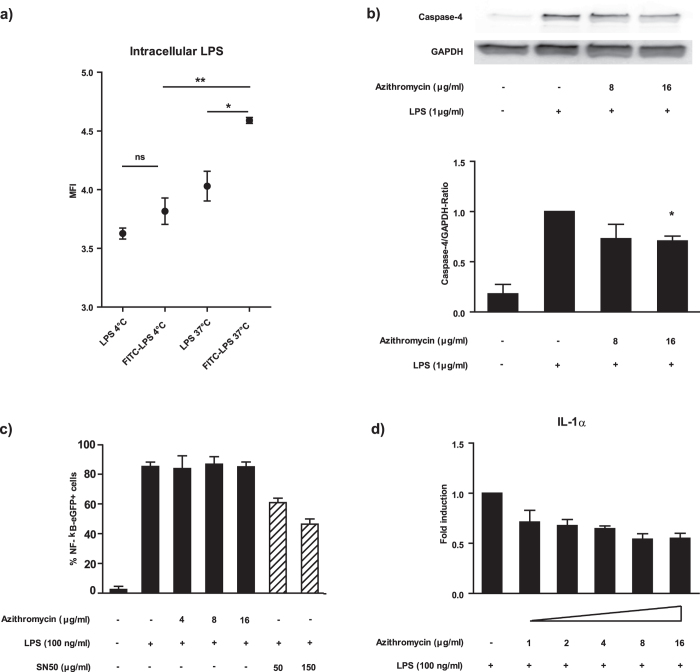
Azithromycin inhibits the induction of caspase-4 without affecting NF-κB transcription. Panel a) depicts the intracellular accumulation of FITC-labelled LPS at 37 °C into monocytes after 4 h incubation. Cells incubated at 4 °C were included as controls. Quenching with trypan blue was performed to exclude extracellular fluorescence. A representative of 2 independent experiments performed in triplicates is shown. Panel b) depicts a representative immunoblot result and the summary of the densitometric analysis of 3 independent experiments of caspase-4 induction in azithromycin treated and LPS stimulated human monocytes. Panel c) shows the impact of azithromycin and the NF-κB inhibitory protein SN50 (positive control) on LPS induced NF-κB transcription in an eGFP-NF-κB THP-1 monocyte reporter cell line. Panel d) shows the impact of azithromycin on IL-1α release of LPS stimulated human monocytes. Cells were treated with the indicated concentrations of azithromycin and stimulated with 100 ng/ml LPS for 20 h. Supernatants were analysed for the presence of IL-1α by Luminex^®^. Values in Panel a) are expressed as Mean ± SEM of triplicate values of a representative experiment.*Significant compared to the indicated samples, *p < 0.05, **p < 0.01 calculated by unpaired t-test. b)+c)+d) are expressed as mean ± SEM of 3 independent experiments. *Significant compared to LPS alone, p < 0.05 calculated by paired t-test.

The finding that azithromycin inhibits IL-1β secretion upon LPS stimulation without affecting the production of other pro-inflammatory cytokines, suggested an inhibitory effect of the drug on IL-1β maturation, since the first step of cytokine induction (NF-κB activation) would also affect the production of other cytokines. Furthermore, the restriction of the effect to LPS stimulation suggested that the mechanism either included the NLRP3 inflammasome or directly involved LPS recognition. In order to assess whether a disturbance of intracellular LPS sensing might be responsible for azithromycin’s effects, we measured whether there is an uptake of LPS into intracellular compartments of human monocytes in the doses utilised in our cytokine assays. To this end, we made use of FITC-labelled LPS to detect internalisation of this compound after quenching of extracellular fluorescence. Here, we were able to detect a significant increase in fluorescence in FITC-LPS treated cells, when compared to both LPS-stimulated (37 °C) and/or FITC-LPS treated (4 °C) cultures, thus indicating internalisation of the compound (Fig. 4a). Since we had detected intracellular LPS, a disturbance of LPS sensing by azithromycin was a feasible option. Inflammatory caspase-4, a human homologue of murine caspase-11, has recently been identified to function as an intracellular LPS receptor^14^. When analysing the impact of azithromycin on the induction of this caspase, we found a significant down-regulation after treatment (Fig. 4b, upper and lower panel). As expected, NF-κB signalling was not affected by azithromycin treatment (Fig. 4c). Once we identified that azithromycin’s effects were mediated by interference with inflammatory caspase signalling, we wondered whether other cytokines regulated by this axis were also affected by azithromycin treatment. Therefore, we analysed the induction of IL-1α and IL-18, and found an intense and dose-dependent down-modulation of IL-1α production upon LPS stimulation (Fig. 4d, Suppl. Table S1). In contrast, we were not able to detect IL-18 in cell culture supernatants (data not shown). With these experiments, we were able to identify an impact of azithromycin on inflammasome formation, which involves the inhibition of intracellular LPS sensing mediated by caspase-4.

### Azithromycin affects IL-1β secretion *in vivo*

**Figure 5:**
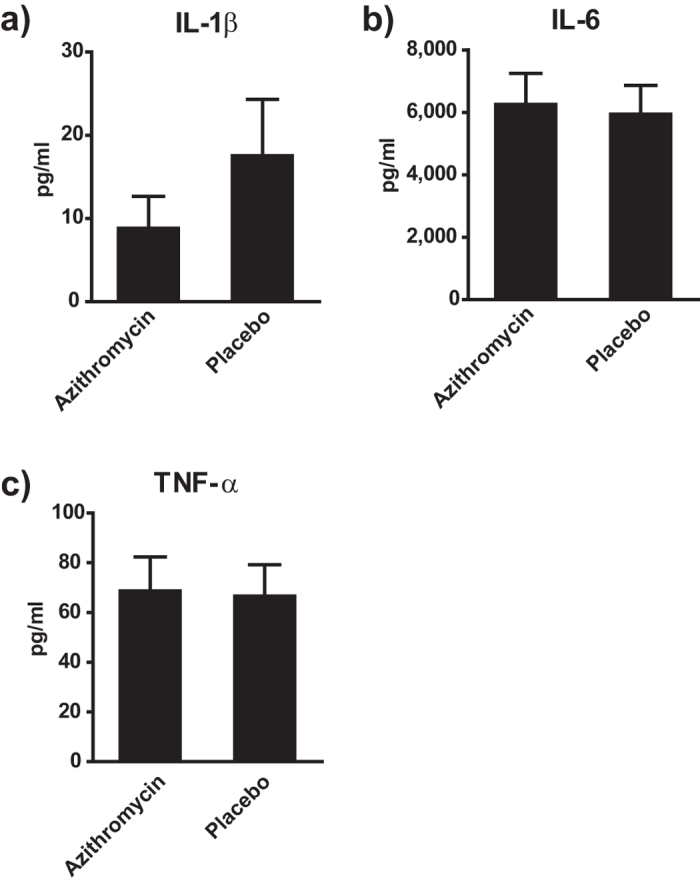
Azithromycin affects IL-1β secretion *in vivo*. Panel a)-c) show the impact of azithromycin treatment on cytokine levels in a murine LPS-sepsis model. Female C57BL/6 (n = 10 mice per group) were injected with 50 mg/kg BW azithromycin or placebo and subsequently were injected with 2.5 mg/kg BW LPS. Serum samples were obtained 6 h after injection and cytokines were measured by Luminex^®^. Results are depicted as mean ± SEM.

In order to further substantiate our findings, we assessed the impact of azithromycin on cytokine induction in a murine model of LPS sepsis. In line with our *in vitro* observations, we found a selective down-modulation of IL-1β levels in the course of endotoxin sepsis whereas other pro-inflammatory cytokines remained unaffected by the treatment (Fig. 5a–c). Although the results did not reach statistical significance, probably due to the high variability in this model, the specificity of azithromycin’s effects on cytokine production is remarkable. These findings substantiate the potential impact of our findings for an *in vivo* situation which might be of relevance in clinical conditions as well.

## Discussion

Several clinical studies addressing the impact of macrolides on inflammation-driven diseases found a therapeutic benefit of macrolides which was not exclusively attributable to their antimicrobial properties^3^,^6^,^9^. These findings have stimulated a debate about whether a long-term macrolide regimen should become part of treatment guidelines in patients with chronic respiratory diseases such as COPD and cystic fibrosis^32^,^33^.

The aim of this study was to analyse and compare the impact of macrolides on the inflammasome axis, which was recently identified as a central regulator of respiratory diseases, a major field of clinical macrolide application. We have shown that azithromycin selectively inhibits IL-1 production *in vitro* and *in vivo*. Azithromycin did not affect NF-κB signalling in monocytes, but impaired the induction of the inflammatory caspase-4, which is an early regulator of the inflammasome pathway and an intracellular LPS sensor. In contrast, clarithromycin and roxithromycin did not exhibit any cytokine modulating effects in human monocytes. Experiments interfering with Ca^++^-dependent uptake indicated a role of intracellular accumulation of azithromycin in its cytokine-modulatory effects.

Although a plethora of work analysing the immune-modulating properties of macrolides has been performed^1^, studies on the cytokine-modulating properties of these drugs on primary human innate immune cells are limited. Similar to our results, Vrančić *et al.* found no alterations of TNF-α and IL-6 production of IFN-γ/LPS stimulated human monocytes when co-treated with azithromycin^34^. Kikuchi *et al.* studied the impact of clarithromycin on IL-8 secretion of adherence-purified monocytes and the THP-1 monocytic cell line. In contrast to our findings, these authors were able to detect inhibition of IL-8 secretion. Since we utilized highly purified CD14^+^ cells, the inconsistency might be explained by the different cell isolation procedures and cell treatment. Furthermore, Kikuchi *et al.* used higher amounts of LPS (1 μg/ml vs 100 ng/ml in our study) for stimulation, which might influence the outcome as well^35^. The only other study assessing potential differences in the cytokine-modulating effects of azithromycin and clarithromycin in primary human monocytes was performed by Khan *et al.*^36^. Similar to our findings, differing modulating properties of the drugs were observed. However, in this study, slight reductions also in IL-6 and TNF-α levels were observed when the cells were treated with clarithromycin. Intriguingly, similar to our results, azithromycin potently inhibited IL-1α secretion in this study. Nevertheless, since the regulation of IL-1 secretion by the inflammasome axis was then unknown, the inflammasome could not be deduced to be the molecular target of azithromycin.

Macrolides are known to accumulate in leucocytes, and their intracellular concentrations are about 100-fold higher than that in plasma^37^. The doses utilized in this study mirror tissue concentrations determined by pharmacokinetic studies which range from 5 to 10 μg/ml^38^. Azithromycin is known to accumulate intracellularly to a much higher extent than clarithromycin or roxithromycin^28^,^29^. The dibasic structure of azithromycin, which is unique among macrolides, causes enrichment in acidic phagosomal compartments due to ion-trapping and a rather slow release from intracellular compartments^39^. Transport through the plasma membrane is known to be mediated by a Ca^++^-dependent mechanism^31^. In this study, we have utilised two strategies known to inhibit macrolide accumulation in leucocytes^30^,^31^. The observation that interruption of azithromycin’s transmembrane transport can abolish its IL-1β modulating effects suggests a role of azithromycin’s intracellular accumulation in the mediation of its effects. Taking into account the established differences in intracellular accumulation among macrolides, the varying ability of macrolides to inhibit the inflammasome axis due to differential intracellular presence is conceivable. Nevertheless, additional experiments are needed to further elucidate the mechanisms underlying the diverging immune-modulatory properties of macrolides.

Several molecular targets have been implicated in azithromycin’s immune-modulating effects^1^. However, our observations in human monocytes narrowed down the spectrum of potential targets to the pathways involved in IL-1β secretion, NF-κB- and inflammasome signalling.

In contrast to previous reports on azithromycin’s impact on the NF-κB pathway in monocytes^34^,^40^, we were not able to detect an inhibition of NF-κB signalling in our model. Since previous reports utilised dual stimulation models in their assays (Vrančić *et al.* utilised an IFN-γ/LPS-stimulation model^34^, Kobayashi *et al.* PMA priming prior to LPS stimulation^40^), varying effects might be due to differences in signalling cascades induced in the respective models.

Recent advances have identified the inflammatory caspase-4 and the murine homologue caspase-11 as central upstream regulators of the “noncanonical inflammasome” cascade^41^. These caspases were found to be crucial regulators of endotoxin-induced immune responses. In particular, they mediate LPS-induced lethality in endotoxin sepsis^13^,^42^. Sollberger *et al.* elegantly showed that caspase-4 is an upstream regulator of caspase-1 activity, and, additionally, has intrinsic IL-1β processing abilities^43^. The findings by Shi *et al.*, who identified caspase-4 as a direct receptor for LPS, shed further light on the role of this enzyme in the mediation of endotoxin-induced immune responses^14^. Since azithromycin’s effects were observed after endotoxin, but not flagellin stimulation, we focused on this inflammatory caspase in the search for azithromycin’s target in inflammasome activation. We were able to show an impairment of caspase-4 induction after azithromycin treatment, which provides a coherent explanation for the specificity of its effects. Additionally, we found a dose-dependent down-modulation of another caspase-processed cytokine, IL-1α, which provides further evidence for azithromycin’s impact on this pathway. Although the induction of IL-1β maturation by caspase-4 is consistent in literature, the involvement of downstream mediators of this effect, particularly caspase-1, is controversial^42^,^43^. Future studies might clarify this issue and help to further elucidate the detailed mechanism of azithromycin’s action.

The specific inhibition of IL-β production by azithromycin in a murine endotoxin sepsis model, points towards a similar effect on caspase-11, the murine homologue of human caspase-4. In the setting of endotoxin sepsis, these inflammatory caspases are not only known to mediate IL-1β secretion, but also, and more importantly, are crucial regulators of lethality in endotoxemia^13^,^42^. The accordance of our *in vitro* data with the findings in this *in vivo* model expands our observations to pathologically relevant situations and might encourage research on the impact of azithromycin on this axis in similar conditions in humans. Furthermore, clinical research needs to assess whether our observations gained in a short term stimulation model are reproducible in long-term treatment regimens with different macrolides in the therapy of clinical conditions.

We herewith provide the first evidence of azithromycin’s specific inhibition of the pro-inflammatory cytokines IL-1α and IL-1β in human innate immune cells by impairing intracellular LPS sensing of the inflammatory caspase-4. Furthermore, we have shown that there are fundamental differences between macrolides in targeting the inflammasome axis. Therefore, our findings suggest that azithromycin might be the macrolide of choice in the treatment of diseases involving deregulated inflammasome activation. However, since this pathway plays an important role in microbial clearance as well, additional studies are required in order to assess whether the immune-modulating properties reported in this study result in a favourable disease outcome.

## Materials and Methods

### Ethical considerations, cell isolation and culture

This study was approved by the local ethics committee of the Medical University of Vienna (EC number 1381/2014) and conducted according to the Declaration of Helsinki and Good Scientific Practice guidelines. Buffy-coats from healthy donors were obtained from the Red Cross Austria. Informed consent was obtained from all volunteers prior to donation. Monocyte isolation was performed as previously described^44^. In brief, peripheral blood mononuclear cells (PBMCs) were obtained by density-gradient separation, utilizing endotoxin-free Lymphoprep (Nycomed Pharma AS, Oslo, Norway). Subsequently, the CD14^+^ monocyte fraction was isolated by MACS^®^ separation according to the manufacturer’s protocol (Miltenyi Biotec, Bergisch Gladbach, Germany). After magnetic cell sorting, the purity of the cells was assessed by flow cytometry (commonly >99%). Monocytes were then washed once in phosphate-buffered saline (PBS; PAA, Pasching, Austria) and resuspended in RPMI 1640 (PAA) containing 10% fetal calf serum (PAA) and antibiotics (100 IU/ml penicillin and 100 μg/ml streptomycin; Sigma-Aldrich, St. Louis, MO).

### Cytokine release assay

Monocytes were resuspended at a density of 1 × 10^6^/ml in medium as described above. The cell suspension was seeded on 96-well plates, and azithromycin, clarithromycin, or roxithromycin (all obtained from Sigma) were added in the indicated concentrations just before stimulation with either 100 ng/ml LPS 0111:B4 (L-4391, Sigma) or 100 ng/ml flagellin (Invivogen, San Diego, CA). After 18–20 h incubation, the supernatants were collected, centrifuged for 5 min at 400 × g and used for further analysis. In the Ca^++^-depleting experiments, cells were treated as described above but were stimulated in medium containing 10 mM EGTA (Sigma). In the Ca^++^-channel blocking experiments, cells were incubated with 125 μM verapamil (Sigma) for 30 min before treatment with azithromycin and LPS stimulation (verapamil was not removed during stimulation). Cytokine measurements of TNF-α, IL-1β, IL-1α, IL-6, and IL-8, were performed by Luminex^®^ testing using specific matched-pair antibodies and recombinant cytokines as standards (Merck Millipore, Billerica, MA). IL-18 was measured with a human IL-18 Instant ELISA (eBioscience, Vienna, Austria; detection limit: 9.2 pg/ml).

### Cell viability assay

Monocytes were treated as described for the cytokine release assay. After 20 h co-incubation with LPS and azithromycin, cells were detached by washing with PBS/4 mM EDTA, washed once in PBS and resuspended in 25 μl PBS/EDTA. Shortly before performing flow cytometric analysis, propidium iodide was added to a final concentration of 50 ng/ml.

### Intracellular LPS measurement

Monocytes (1 × 10^6^/ml) were incubated with either unlabelled LPS 0111:B4 (Sigma) or FITC-labelled LPS 0111:B4 (Sigma) at 37 °C for 4 h, baseline controls incubated at 4 °C were included in the analysis. After the incubation period, cells were detached with Accutase (PAA), washed with PBS and quenching was performed with trypan blue (Glycotope Biotechnology GmbH, Heidelberg, Germany) before flow cytometric analysis on a BD LSRFortessa™ (Becton Dickinson, Franklin Lakes, NJ).

### Western blot analysis

Monocytes (1 × 10^6^/ml) were incubated with azithromycin in the indicated concentrations before adding 1 μg/ml LPS 0111:B4. After 18 h of incubation, the cells were detached by scraping, washed in ice-cold PBS, and lysed in 0.5% Triton-X buffer for 5 min on ice. After lysis, the suspension was centrifuged for 5 min at 13000 × g, and the supernatant was utilized for further analysis. Western blotting was performed as described previously^44^. Anti-caspase-4 antibody (MBL International, Woburn, MA; clone 4B9) and anti-GAPDH (Cell Signaling Technology, Danvers, MA) were used in a dilution of 1:1000. Detection was performed with Pierce® ECL Western blotting substrate (Thermo Fisher Scientific, Waltham, MA) on a LAS-4000 (Fujifilm, Tokyo, Japan). Data analysis, quantification, and processing were performed with Fiji (ImageJ) image processing software.

### NF-κB-GFP reporter cell assay

For the generation of THP-1-NF-kB-GFP reporter cells, a slightly modified form of a previously described GFP-NF-kB reporter construct^45^ was retrovirally transduced in THP-1 cells (ATCC #TIB-202). Single cell clones were established by limiting dilution culturing. A cell clone that was GFP-negative in an unstimulated state and strongly upregulated GFP expression upon LPS or PMA/ionomycin treatment was selected for further use.

The reporter cells were incubated with the indicated amounts of azithromycin, LPS and SN50 (Enzo Life Sciences, Inc.; Farmingdale, NY) for 20 h before flow cytometric analysis of eGFP fluorescence on a BD LSRFortessa™.

### Murine LPS-sepsis

All animal experiments were reviewed by the Animal Ethics Board of the Medical University of Vienna and approved by the Austrian Ministry of Economy and Science (BMWF-66.009-0027-II/3b/2014). All animal husbandry and experimentation was performed in accordance with the Federation of Laboratory Animal Science Associations (FELASA) guidelines and national law.

Eight-week-old female C57BL/6 mice (10 mice per group) from in-house breeding (originally obtained from Jackson Laboratories, Bar Harbor, ME) were used for the experiment. Mice were injected intraperitoneally with 50 mg/kg body weight (BW) azithromycin (Sigma) or placebo (DMSO as solvent control) before receiving an injection of 2.5 mg/kg BW LPS 0111:B4 (L-2630, Sigma). After 6 h, blood samples were obtained from tail vein puncture, and the serum was centrifuged and subjected to cytokine analysis by Luminex^®^ (as above).

## Additional Information

**How to cite this article**: Gualdoni, G. A. *et al.* Azithromycin inhibits IL-1 secretion and non-canonical inflammasome activation. *Sci. Rep.* **5**, 12016; doi: 10.1038/srep12016 (2015).

## Supplementary Material

Supplementary Information
