# Supplementary material for: Azithromycin inhibits IL-1 secretion and non-canonical inflammasome activation
Source: Sci Rep. 2015 Jul 8;5:12016. doi: 10.1038/srep12016 (PMC4495566; doi:10.1038/srep12016)
Supplement: Supplementary Information [file srep12016-s1.pdf]

Supplement

Supplementary Information

Azithromycin inhibits IL-1 secretion and non-canonical inflammasome activation

Guido A. Gualdoni[1], Tilman Lingscheid[2], Klaus G. Schmetterer[3], Annika Hennig[1], Peter Steinberger[1], Gerhard J. Zlabinger[1*]

[1]Institute of Immunology, Center for Pathophysiology, Infectiology and Immunology, Medical University of Vienna, Vienna, Austria

[2]Department of Infectious Diseases and Pulmonary Medicine, Charité - Universitätsmedizin Berlin, Germany

[3]Department of Laboratory Medicine, Medical University of Vienna, Vienna, Austria

*Corresponding author: Gerhard J. Zlabinger, Institute of Immunology, Medical University of Vienna, Vienna, Austria. Tel: +43-1-40160-33271, Fax: +43-1-40160-933213, email: gerhard.zlabinger@meduniwien.ac.at

Supplement

Supplementary Table S1

Cytokines	LPS	FLA
Azithromycin Cytokine Assays (Fig. 1, Fig. 4)		
IL-1β	3.31 ng (±1.32), Median: 1.64 ng	1.05 ng (±0.38), Median: 0.95 ng
IL-6	8.15 ng (±2.71), Median: 5.46 ng	5.20 ng (±0.31), Median: 4.80 ng
IL-8	10.26 ng (±2.01), Median: 10.22 ng	9.20 ng (±1.63), Median: 7.92 ng
TNF-α	4.84 ng (±1.01), Median: 5.18 ng	3.92 ng (±0.88), Median: 4.26 ng
IL-1α	0.51 ng (±0.27), Median: 0.44 ng	
Clarithromycin/Roxithromycin Cytokine Assays (Fig. 2, Suppl. Fig. S2)		
IL-1β	1.32 ng (±0.10), Median: 1.31	0.81 ng (±0.46), Median: 0.35 ng
IL-6	10.78 ng (±4.80), Median: 6.82	9.62 ng (±5.56), Median: 4.62 ng
IL-8	9.17 ng (±4.39), Median: 10.22	7.67 ng (±4.12), Median: 6.41 ng
TNF-α	2.94 ng (±0.81), Median: 3.41	1.99 ng (±1.11), Median: 1.22 ng
Ca^{++}-interference experiments (Fig.3)		
IL-1β (EGTA-treated)	3.32 ng (±1,59), Median: 3.39 ng	
IL-1β (Verapamil-treated)	2.18 ng (±0.69), Median: 1.84 ng	

Suppl. Table S1. Cytokine levels after LPS and flagellin stimulation of human monocytes. Values are expressed as mean ±SEM and median of 3-6 independent experiments.

Supplement

Supplementary Figure S1

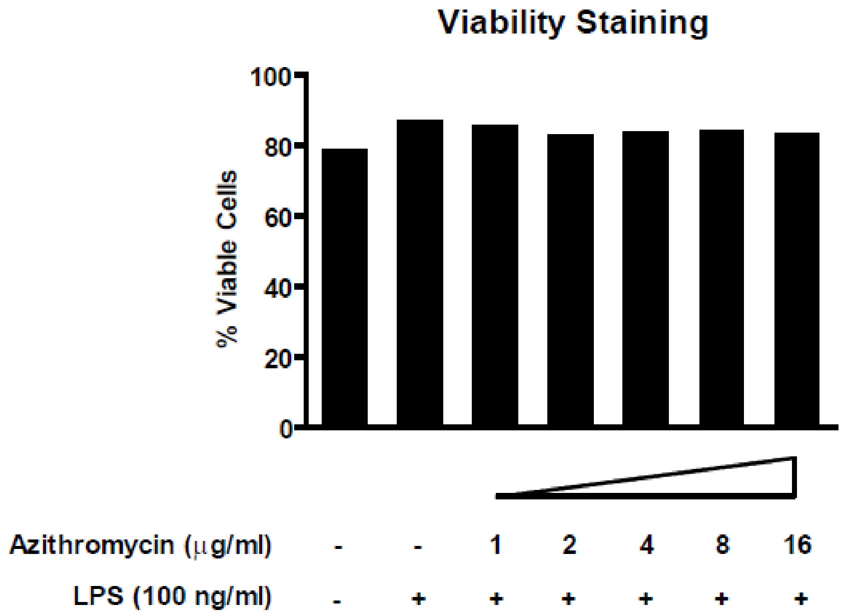

Suppl. Figure S1. Azithromycin has no cytotoxic effect upon human monocytes. Human monocytes were treated with the indicated concentrations of azithromycin and 100 ng/ml LPS. After 20 h the supernatants were harvested and cytokine measurements were performed. Adherent cells were then detached, washed in PBS and propidium iodide was added just before flow cytometric analysis.

Supplement

Supplementary Figure S2

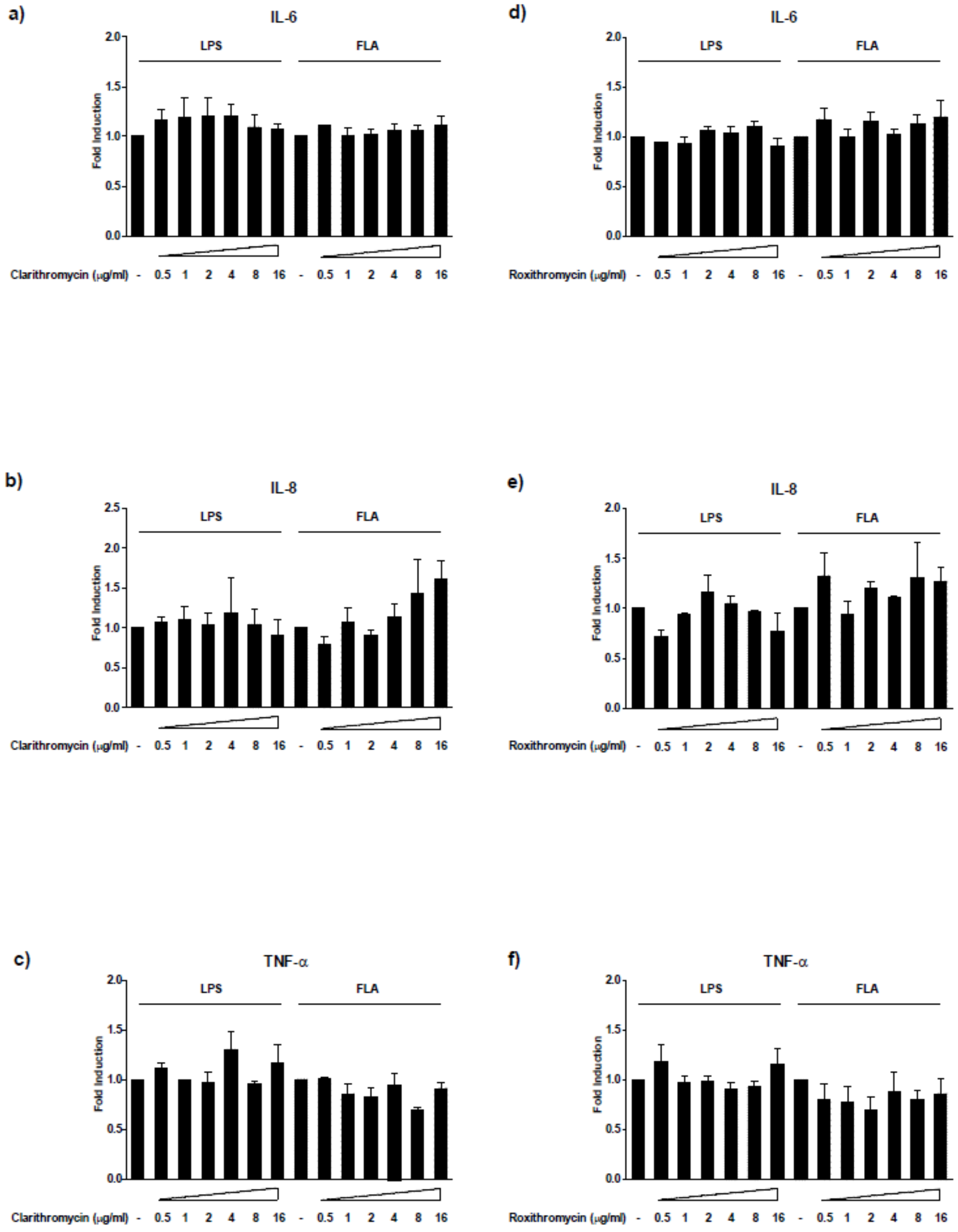

Suppl. Figure S2. Clarithromycin and roxithromycin have no impact on cytokine release of LPS or flagellin stimulated monocytes. Panel a)-c) and d)-f) show the impact of clarithromycin and roxithromycin, respectively, on LPS or flagellin stimulated cytokine release of human monocytes. Cells were treated with the indicated concentrations of the antibiotics and stimulated with either 100 ng/ml LPS or 100 ng/ml flagellin for 20 h. After co-incubation, cell culture supernatants were harvested and analysed for the presence of the indicated cytokines by Luminex®. Values are expressed as mean ± SEM from 4 independent experiments.
